# Distributional coding of associative learning in discrete populations of midbrain dopamine neurons

**DOI:** 10.1016/j.celrep.2024.114080

**Published:** 2024-04-04

**Authors:** Riccardo Avvisati, Anna-Kristin Kaufmann, Callum J. Young, Gabriella E. Portlock, Sophie Cancemi, Rui Ponte Costa, Peter J. Magill, Paul D. Dodson

**Affiliations:** 1 School of Physiology, Pharmacology, and Neuroscience, University of Bristol, BS8 1TD, UK; 2 Medical Research Council Brain Network Dynamics Unit, Nuffield Department of Clinical Neurosciences, University of Oxford, OX1 3TH, UK; 3 Computational Neuroscience Unit, Department of Computer Science, SCEEM, Faculty of Engineering, University of Bristol, BS8 1UB, UK

## Abstract

Midbrain dopamine neurons are thought to play key roles in learning by conveying the difference between expected and actual outcomes. Recent evidence suggests diversity in dopamine signaling, yet it remains poorly understood how heterogeneous signals might be organized to facilitate the role of downstream circuits mediating distinct aspects of behavior. Here we investigated the organizational logic of dopaminergic signaling by recording and labeling individual midbrain dopamine neurons during associative behavior. Our findings show that reward information and behavioral parameters are not only heterogeneously encoded, but also differentially distributed across populations of dopamine neurons. Retrograde tracing and fiber photometry suggest that populations of dopamine neurons projecting to different striatal regions convey distinct signals. These data, supported by computational modelling, indicate that such distributional coding can maximize dynamic range and tailor dopamine signals to facilitate specialized roles of different striatal regions.

## Introduction

Learning to anticipate positive or negative consequences from environmental cues is essential for survival. Midbrain dopamine neurons are thought to play key roles in this process by signaling the difference between expected and actual outcomes (reward prediction error; RPE)^1^. Such a fundamental teaching signal has traditionally been thought to necessitate a uniform message^2–4^. However, recent work has revealed that dopamine neurons and the signals they convey might be heterogeneous. For example, differences in dopamine release and dopamine axon activity have been observed in different striatal regions^5–11^ with temporally-distinct activity patterns recorded across dorsal striatum^12^. Furthermore, dopamine neurons seem to signal more than just reward; encoding movement onset, movement kinematics, and multiple variables involved in decision-making^13–20^. Dopamine neurons also seem to be molecularly, physiologically, and anatomically diverse; single-cell RNA sequencing has identified multiple groups of midbrain dopamine neurons that can be distinguished by the combinatorial expression of different molecular markers^21–27^, and there is evidence that dopamine neurons exhibit differences in ion channels, other protein expression, firing properties, and input-output connectivity^21,22,26,28–35^. Together, these findings suggest that there might be functionally-specialized midbrain populations that each convey different information to discrete brain areas. However, it is not yet clear how such heterogenous signals might instruct different striatal regions, which themselves have diverse and complimentary functional/behavioral roles. For example, the dorsolateral striatum (DLS) is thought to subserve sensorimotor functions in stimulus-response associations and habits, whereas the dorsomedial striatum (DMS) plays roles in response-outcome associations for goal-directed actions^36^. In further contrast, the ventrolateral striatum (VLS) is thought to be important for motivation^37^, whereas the core of the nucleus accumbens (NAc) has been ascribed roles in outcome evaluation^8,38^.

To define the organizational principles underlying heterogeneous dopamine signaling, we recorded and molecularly-identified individual dopamine neurons in mice during Pavlovian conditioned behavior. We find that there is no broad spatial organization of encoding in midbrain, but instead we show that neurons likely projecting to the same target are more homogenous in their firing patterns and these patterns match the activity of dopamine axons in the striatal target region. Temporal difference modelling predicts that distributional coding within these populations can tailor them to support different aspects of associative learning.

## Results

**Figure 1:**
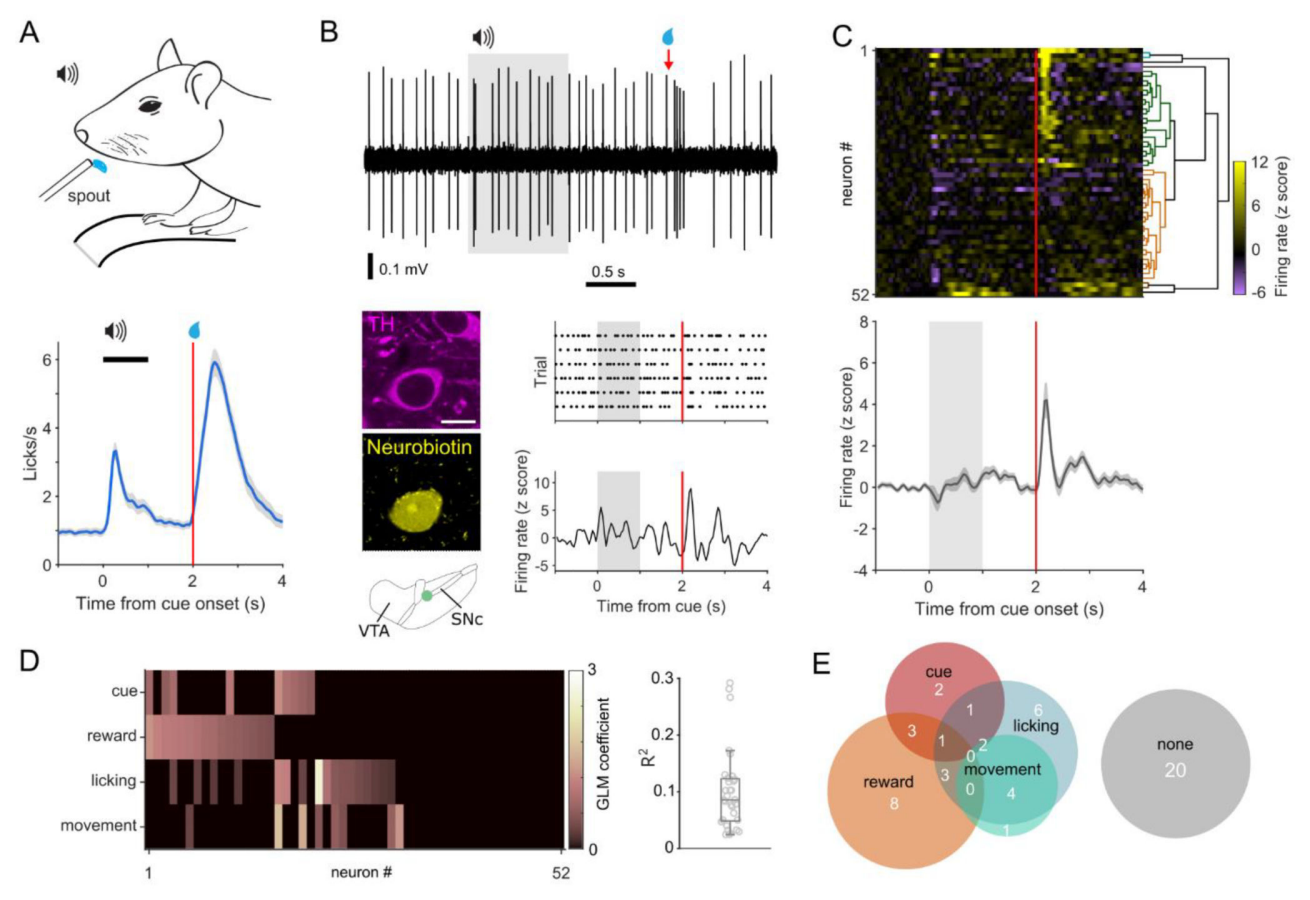
Dopamine neurons heterogeneously encode reward and predictive cues. (A) Head-fixed mice, positioned on a treadmill, were trained to associate a 4 kHz auditory tone (cue) with delivery of reward. After training, mice show robust anticipatory licking after the cue and licking to receive reward. (B) Extracellular recording of action potential firing (*top*) and corresponding peri-stimulus time histogram (PSTH; lower right) from an individual dopamine neuron during Pavlovian conditioned behavior. Grey shading indicates cue duration, red line indicates reward delivery. After recording, individual neurons were juxtacellularly labeled with neurobiotin and tested for immunoreactivity to tyrosine hydroxylase (TH: lower left; scale = 10 μm) to confirm their dopaminergic identity and localization in the midbrain. The schematic depicts the location of the neuron in the dopaminergic midbrain. (C) Z-scored PSTH of individual responses from identified dopamine neurons (*rows*) grouped by hierarchical clustering (*top*), and mean response (*bottom*). (D) Features which correlate with changes in firing rate for each neuron, determined by a general linear model (GLM). (E) GLM output suggests that some neurons multiplex signals by encoding multiple aspects of behavior (numbers indicate the proportion of neurons; note that some combinations are unable to be displayed). N = 52 neurons from 30 mice.

We trained head-fixed mice in a Pavlovian conditioning paradigm in which an auditory cue (1 s, 4 kHz tone) signaled reward delivery after a fixed delay (2 s from cue onset). Mice rapidly learned to associate the cue with reward as indicated by anticipatory licking during cues (Figure 1A and Figure S1). To investigate the firing of different dopamine neurons once mice had learnt the association, we extracellularly recorded individual neurons and then juxtacellularly labeled them to precisely determine their location and confirm they were dopaminergic (Figure 1B).

### Dopamine neurons heterogeneously encode reward behavior

The majority of dopamine neurons altered their firing rate at the onset of the cue and/or reward (Figure 1B and C). However, while changes at reward were generally increases in firing, changes at cue onset were a mix of increases and decreases in rate. Firing at cue and reward may reflect the encoding of reward prediction^39^, or alternatively, encoding of actions with which to obtain reward^15,18,19,40^. To investigate whether changes in firing rate correlated with cue, reward, licking, or other kinds of movement (e.g. walking, running, or postural adjustments), we used a general linear model (GLM) (Figure 1D, 1E, and Figure S2) to investigate behavior-related firing across the session. We found that many neurons encoded reward (16 of 52) and/or cue (9 of 52). However, we also found that a significant number of neurons encoded parameters which were not obvious from the peristimulus time histogram, including licking (17 of 52) or movement (8 of 52). A considerable proportion of neurons (20 of 52) did not significantly encode (p > 0.05) any of the features we examined (Figure 1D and 1E), which suggests that there may be populations of dopamine neurons that encode other facets of behavior, or that these neurons show no response to the fully predicted reward. Interestingly, many neurons encoded more than one parameter (13 of the 32 neurons encoding behavioral parameters), concordant with the idea that dopamine neurons multiplex signals^16,20^. These data suggest that there is not a uniform signal across midbrain dopamine neurons, but instead support a framework of heterogeneity with some neurons encoding single parameters and others multiplexing signals to encode several distinct aspects of behavior (Figure 1E).

### Encoding is not clearly defined by anatomical location

**Figure 2:**
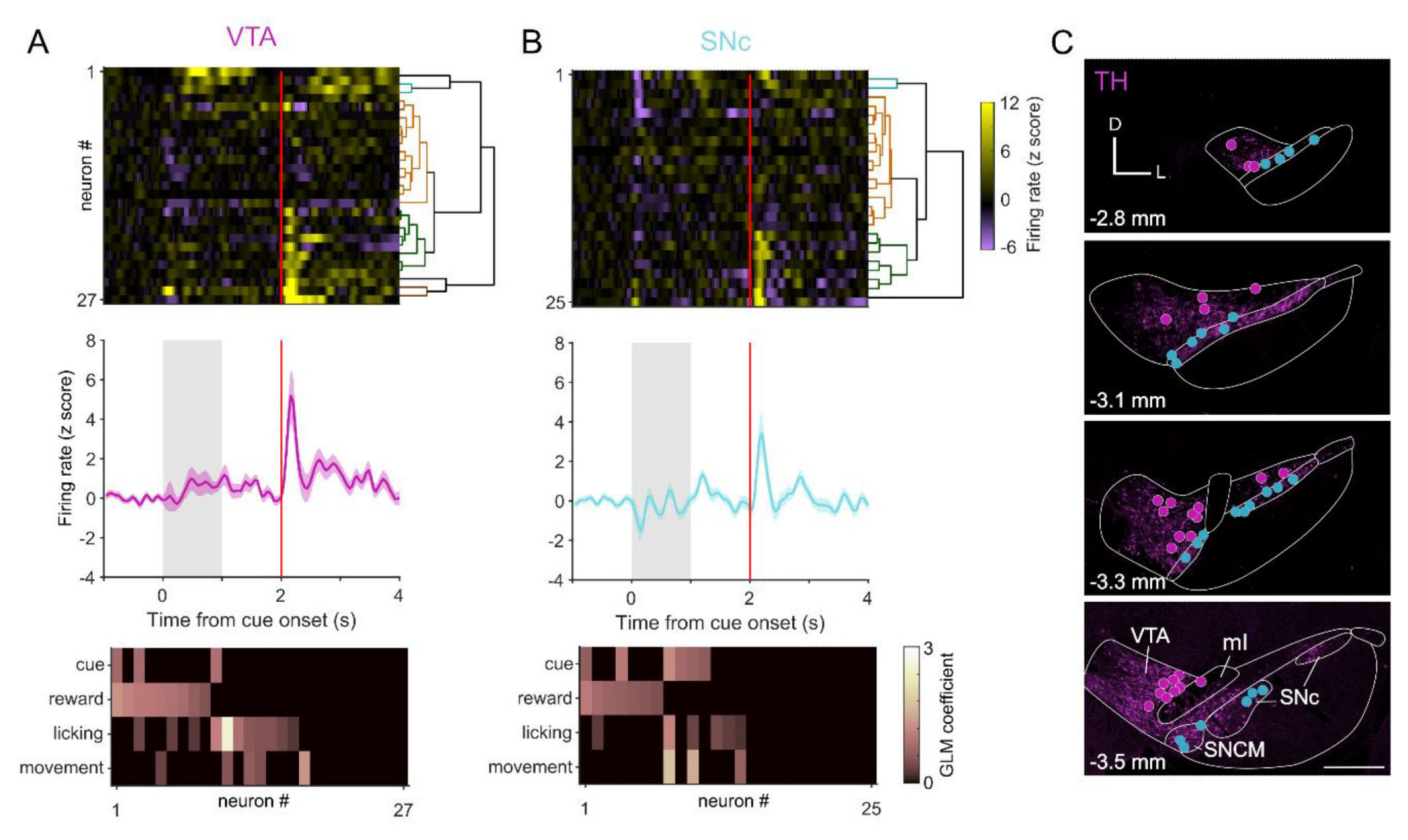
Anatomical subgroups do not account for response heterogeneity. (A) VTA neurons heterogeneously encoded aspects of Pavlovian conditioned behavior (top) and firing could be classified as encoding multiple parameters by GLM (bottom). (B) SNc neurons exhibit a similar degree of heterogeneity in firing responses. (C) Schematic of the locations of recorded and juxtacellularly labeled neurons (VTA neurons magenta, SNc cyan) overlayed on example images of tyrosine hydroxylase (TH) immunofluorescence at four anteroposterior positions 2.8 to 3.5 mm from bregma. D = dorsal; L = lateral; VTA = ventral tegmental area; SNc = substantia nigra pars compacta; ml=medial lemniscus; SNCM=medial part of SNc. Scale = 500 μm. N = 52 neurons from 30 mice.

**Figure 3:**
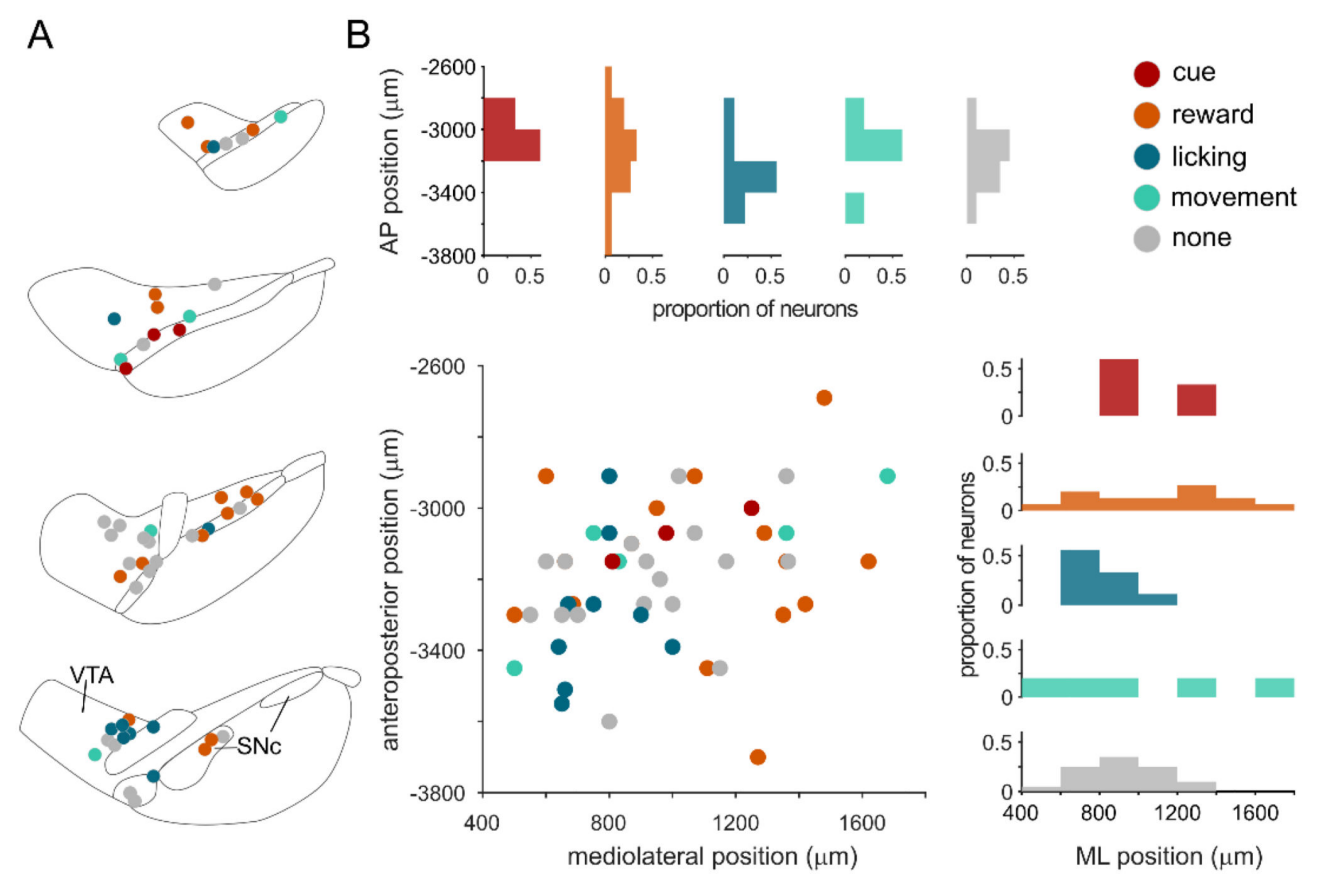
Encoding of behavioral parameters is not focally organized. (A) Schematic of the dominant parameter encoded by each dopamine neuron according to their location in the midbrain. Note some of these neurons will also encode other parameters (e.g. cue in addition to reward). (B) The dominant parameter plotted according to the neurons anteroposterior (AP) or mediolateral (ML) position with respect to bregma. The proportion of neurons encoding each dominant parameter is shown in the AP (top) and ML plane (right). N=52 neurons from 30 mice.

What underlies the heterogeneity we observe in reward-signaling? Given the different roles ascribed to the ventral tegmental area (VTA) and the substantia nigra pars compacta (SNc), one might predict that neurons in these regions would encode the paradigm differently^41–43^. To investigate this possibility, we divided neurons into those located in the VTA (n = 27) or SNc (n = 25) and compared their responses during the session. Despite previous observations of differences in encoding of spontaneous body movements by VTA and SNc neurons^14^, the responses of these two groups during Pavlovian conditioned behavior were nearly identical (Figure 2A–C). The comparable proportions of neurons in each region encoding cue, reward, licking, and movement (Figure 2A and 2B) also suggest that neurons in the two regions encode the paradigm in a similar manner. However, it is possible that such a blunt subdivision may mask finer spatial organization. We therefore considered whether encoding was organized so that neurons in close proximity signaled similar parameters. To probe this possibility, we plotted the dominant parameter (defined by the GLM coefficient) encoded by each neuron in Cartesian space (Figure 3A and 3B). We found that all parameters were represented across the anteroposterior and mediolateral extent, arguing against a precise focal encoding of parameters in different regions of the dopaminergic midbrain.

### Neurons projecting to different striatal regions express alternate combinations of proteins

**Figure 4:**
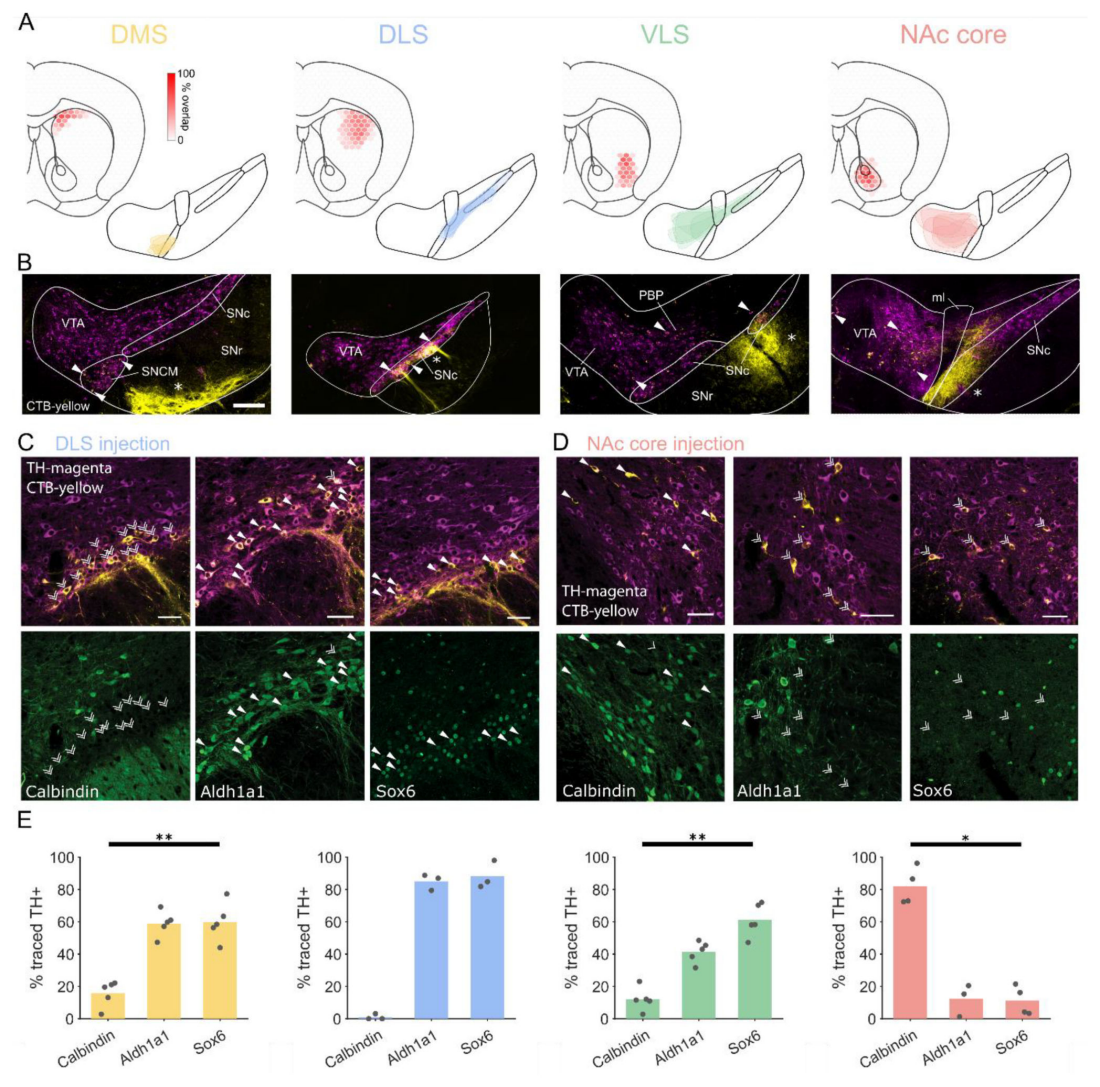
Differential marker expression in populations projecting to different parts of striatum. (A) Schematics showing the extent of retrograde tracer injections into different striatal regions (top left: DMS, dorsomedial striatum; DLS, dorsolateral striatum; VLS, ventrolateral striatum; NAc core, core of the nucleus accumbens) and corresponding boundaries of CTB-labelled dopamine neurons in the midbrain (bottom right). Colored hexagons represent overlap of cholera toxin B (CTB) injections between animals. (B) Representative midbrain images following CTB injection into different striatal regions. Selected retrogradely traced dopamine neurons (filled arrowheads) illustrate the region of the dopaminergic midbrain which projects to each striatal target. Anterogradely traced fibers (asterisks) from striatal projection neurons are also visible in the SNc and SNr. VTA = ventral tegmental area; SNc = substantia nigra pars compacta; SNr = substantia nigra pars reticulata; ml = medial lemniscus; SNCM = medial part of SNc; PBP = parabrachial pigmented area of the VTA. Scale = 100 μm. (C) Retrograde tracing with CTB injected into the DLS identified SNc dopamine neurons (positive for tyrosine hydroxylase, TH; top panels) which innervate this region. The majority of these neurons expressed Aldh1a1 and Sox6, but not calbindin. Filled arrowheads represent marker expression, double arrowheads, lack of expression. Scale = 50 μm. (D) Retrograde tracing in NAc core identified VTA dopamine neurons expressing calbindin, but not Aldh1a1 or Sox6. Scale = 50 μm. (E) Cell counting of CTB positive dopaminergic neurons revealed the percentage of neurons which express each marker. Each data point represents the counts from a single tracer injection. Kruskal-Wallis one-way ANOVA on ranks with Tukey’s post-hoc test; * p < 0.05, ** p < 0.01. N = 17 counts for calbindin and Sox6, 16 for Aldh1a1 from 17 animals.

If encoding is not spatially organized in the midbrain, is there another structural principle underlying response heterogeneity? Emerging evidence suggests that dopamine neurons projecting to particular target regions may differently encode parameters^6,7,30,38^. We therefore considered whether distinct midbrain-striatal circuits may account for some of the heterogeneity we observe. We injected retrograde tracer (cholera toxin B subunit; CTB) into the dorsomedial striatum (DMS), dorsolateral striatum (DLS), ventrolateral striatum (VLS), or nucleus accumbens core (NAc core) and examined the expression of three proteins (Aldh1a1, Sox6, and calbindin) known to be differentially expressed in the dopaminergic midbrain^26^ (Figure 4A). We found that most dopamine neurons projecting to DLS expressed Sox6 and Aldh1a1, but not calbindin, and were located in SNc, whereas those projecting to NAc core were located in VTA and had the opposite expression pattern (i.e. calbindin, but not Aldh1a1 or Sox6; Figure 4B–E). The marker expression of neurons projecting to DMS was less binary, with moderately prevalent expression of Aldh1a1 and Sox6, and rare expression of calbindin (Figure 4E). Notably, we found that these neurons are tightly clustered in the medial part of SNc (SNCM; Figure 4A–B)^34^. VLS-projecting neurons were predominantly localized to the parabrachial pigmented area of the VTA (PBP) and SNc^34^, and expressed Sox6 (Figure 4A–B and 4E). We found a significant interaction between marker expression and region (2 way parametric ANOVA with region and marker as factors; P < 0.0001) suggesting populations can be distinguished using a combination of location and marker expression.

### Dopamine neuron populations encode distinct aspects of behavior

**Figure 5:**
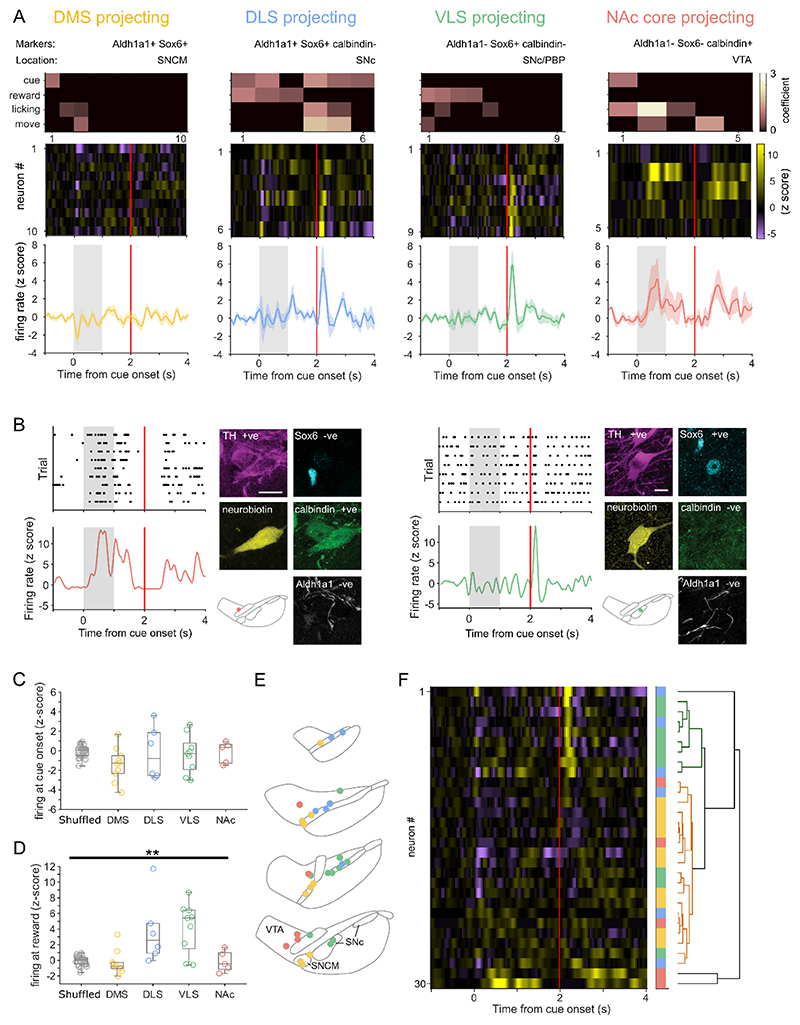
Neurons projecting to different striatal regions exhibit distinct responses. (A) mean PSTH, and individual heatmap plots for neurons putatively projecting to different striatal regions according to classifying marker combinations and location (bottom). Corresponding GLM plots show responses of each neuron to different behavioral parameters (top). (B) PSTH and marker expression of an example putative NAc core-projecting VTA neuron (left) and VLS-projecting SNc neuron (right). Scale = 10 μm. (C) Firing at cue onset (0 to 240 ms after cue onset) for neurons projecting to each striatal region. There were no significant differences between regions or compared to shuffled baseline periods. (D) Mean firing at reward (40 to 240 ms after reward delivery) for neurons projecting to each striatal region. Putative DLS- and VLS-projecting neurons showed significantly higher firing rates than DMS-projecting neurons (P < 0.05 and P < 0.005 respectively) and compared to shuffled baseline periods (P < 0.05 and P < 0.05), Kruskal-Wallis one-way ANOVA on ranks with Tukey’s post-hoc test; ** p < 0.01. (E) Schematic of the location of recorded neurons, color coded by their putative projection-targets. (F) Hierarchically clustered individual responses (colored blocks denote putative projection target). N = 30 neurons from 19 mice.

We next tested whether populations defined by both protein expression and anatomical location showed differential encoding of associative behavior. The activity of recorded dopamine neurons which putatively project to DMS (classified as such by their location in medial SNc and their expression of Aldh1a1 and Sox6; Figure 5E; n= 10 neurons) differed considerably from the population mean (Figure 5A and 1C), with no increase in firing at reward presentation (Figure 5A). The putative DLS-projecting population encoded the cue (Figure 5A and S3) but, in contrast to DMS-projecting neurons, exhibited a mean increase in firing to reward (Figure 5D). The VLS-projecting population did not to change their firing upon cue presentation (despite the anticipatory licking suggesting that the mice registered and learned the predictive value of the cue), but robustly increased their firing shortly after reward presentation (Figure 5A –5D). While VLS-projecting neurons generally had the largest reward-related firing increases, the population was not significantly different from the DLS-projecting population (Figure 5D). Putative NAc core-projecting neurons also showed a distinct response, increasing their firing at both cue and reward. However, rather than being time-locked to the onset of these events, these increases in firing were delayed by a few hundred milliseconds (Figure 5B), coinciding with periods when the mice were still licking. All populations exhibited some degree of multiplexing, but it was prevalent in the DLS-projecting population where two-thirds of the neurons encoded two or more parameters (Figure 5A). Putative DLS- and DMS-projecting populations decreased firing at movement onset, whereas the firing of VLS or NAc core populations did not change (Figure S4). It has recently been observed in anaesthetized mice that neurons projecting to different regions have different firing properties (Farassat et al., 2019). We therefore tested whether the populations we identified had distinct firing in awake mice at rest (i.e. during the ITI, outside of engagement with the paradigm). We found that DMS, DLS, and VLS populations shared similar properties, whereas the NAc core-projecting population had significantly slower firing rates (p < 0.05) and exhibited longer pauses (p < 0.01) (Figure S5). To examine whether these populations could account for the heterogeneity observed across dopamine neurons we performed hierarchical clustering. Clusters were enriched with neurons from a given population, but did not exclusively contain one population (Figure 5F). For example, 75% of neurons in one cluster were putative VLS-projecting neurons but the other VLS-projecting neurons were ascribed to another cluster.

**Figure 6:**
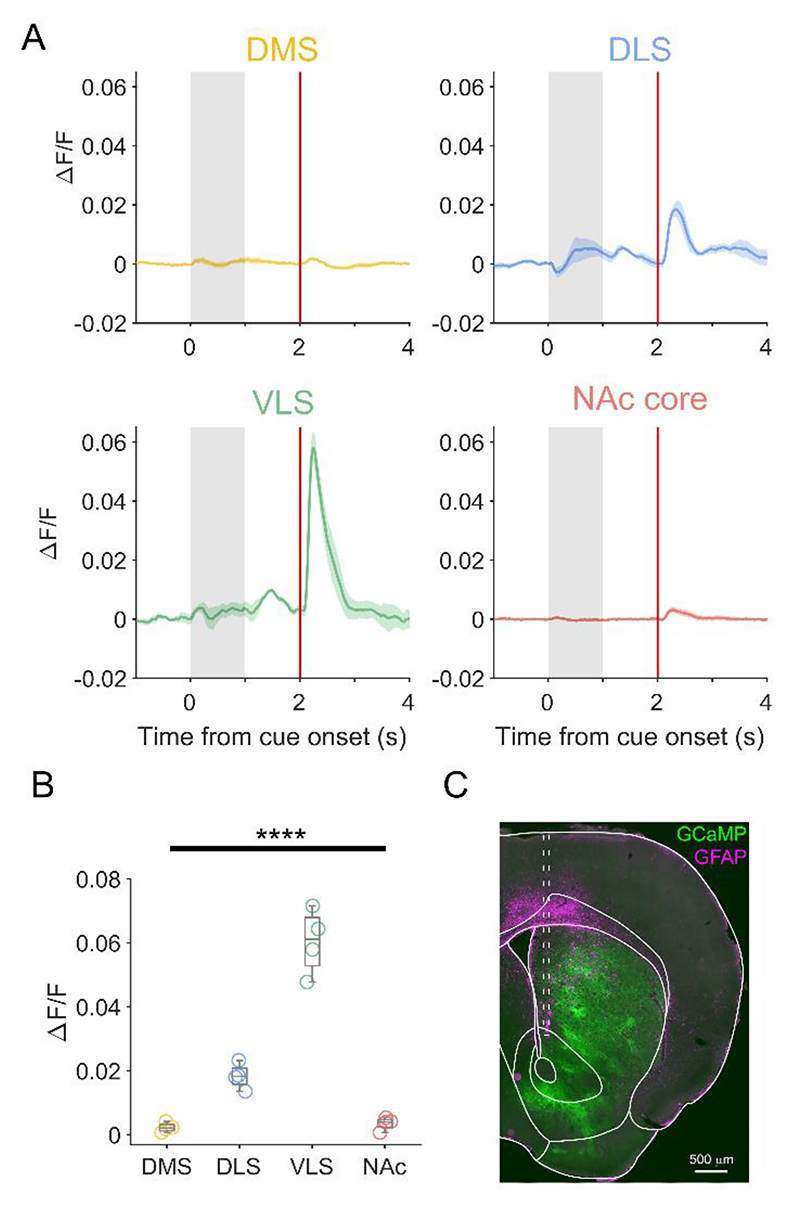
Distinct reward-related signaling in different striatal regions. (A) Mean PSTH of the change in GCaMP6f fluorescence (ΔF/F) in dopaminergic axons in DMS (yellow), DLS (blue), VLS (green), and NAc core (red) recorded with fiber photometry. (B) Mean peak change in fluorescence at reward in each striatal region. DLS and VLS exhibited distinct signals at reward compared to each other population; there was no significant difference between signals in DMS and NAc (P>0.05 one way ANOVA with Tukey’s post-hoc test; **** p < 0.0001). (C) Representative image showing the expression of GCaMP6f and the track (dashed line) of the optic fiber targeted to NAc core. N = 4 recording sessions in 4 mice, (1 recording session per region, per mouse).

**Figure 7:**
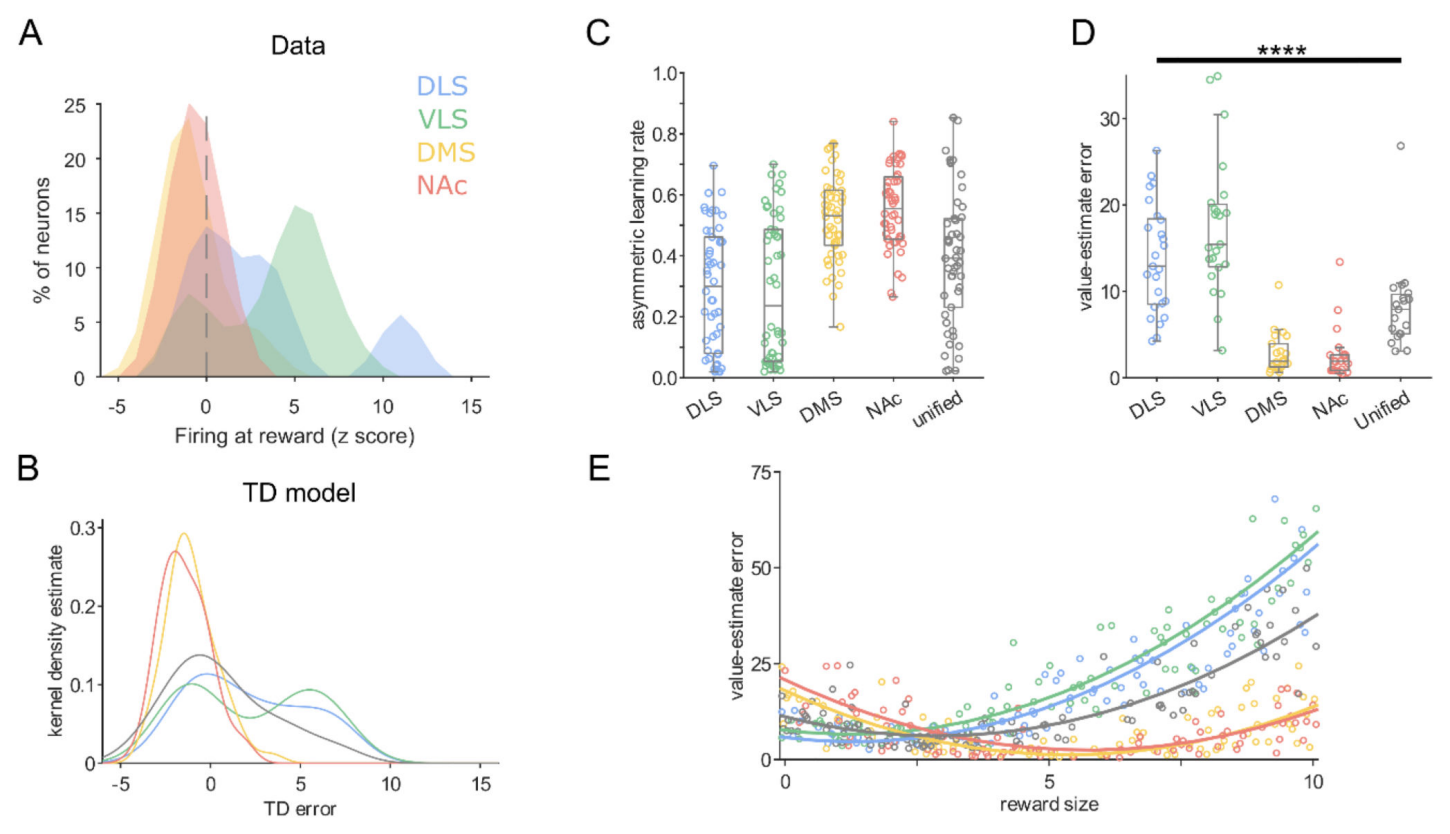
Different distributional coding of reward prediction error supports specialized roles in behavior. (A) Histogram of firing rate at reward for neurons in each putative projection-defined population. (B) Kernel density estimates from the temporal difference (TD) model represent the distribution of TD errors made by the ‘neurons’ comprising each projection-defined agent. The grey line represents a ‘unified population’ generated by sampling from all dopamine neurons in (a). (C) Asymmetric learning rates (α+ / (α+ + α-; balanced learning rates = 0.5) of ‘neurons’ fit from the data in (a) that comprise each agent (N = 50 per agent). (D) The difference between predicted and actual state-values (the mean squared error) to rewards near the center point (reward size of 5) of the training distribution, for each projection-defined agent. DLS- and VLS-projecting populations made significantly worse value-estimates than DMS- or NAc-projecting populations. DMS- and NAc-projecting populations were more accurate than the unified population whereas VLS made significantly larger errors. **** p < 0.0001 Kruskal-Wallis one-way ANOVA on ranks with Tukey’s post-hoc test. (E) Value-estimate errors of each agent for different numerical reward sizes.

If the populations of neurons that we putatively linked to projection targets accurately encompass the neurons projecting to each striatal region, we would predict that not only would the activity of dopamine axons in each target region be distinct, but also that axonal signals will resemble the signals recorded at the soma. To test these predictions, we used acute fiber photometry to record calcium signals in dopaminergic axons in different parts of striatum (Figure 6A–C). We recorded from DMS, NAc core, DLS, and VLS in each mouse (N=4) in different sessions. We observed considerable differences in the activity of dopamine axons in each region, particularly just after reward delivery (Figure 6A). Importantly these signals largely mirrored the action potential firing patterns from our putative projection-defined populations (Figure 5A). Axons in DLS and VLS showed relatively large increases in fluorescence following the fully-predicted reward, whereas axons in DMS and NAc core exhibited negligible changes (Figure 6B); only the DMS/NAc core signals are consistent with models of reward prediction error (RPE)^1^. To probe this further, we examined the normalized firing at reward for each of our putative projection-defined neuronal populations. We found that putative DLS- and VLS-projecting populations had a broader range of responses than DMS- and NAc core-populations suggesting that some neurons were inaccurately estimating future reward (Figure 7A). Recent work has suggested that encoding optimism as a probability distribution across dopamine neurons may confer advantage to reward learning^44^. Our data suggest that distributional coding may differ in populations projecting to different parts of striatum in both the width of the distribution and the skew (Figure 7A).

To explore the potential effect that different distributions might have on reward learning, we used a distributional temporal difference (TD) model where an agent learns state-value associations (Figure 7B). We then tested whether populations putatively projecting to different striatal targets would perform differently compared with a unified population of midbrain dopamine neurons^2–4,44^. Positive and negative learning rates, for an array of neurons, were fit to the juxtacellular data to generate projection-defined agents (Figure 7C). This resulted in each agent having state-value and state-error distributions, with each neuron converging on a different estimate of reward value (Figure 7B). To probe whether different distributions could confer a general advantage, we tested the accuracy of value estimations made by each projection-defined agent (Figure 7D). The model suggests that DMS- and NAc core-projecting populations would make significantly more accurate state-value associations (i.e., smaller value-estimate errors) than a unified population of midbrain dopamine neurons (created from the overall distribution of all recorded neurons; Figure 7D). DLS- and VLS-projecting populations consistently underestimated reward across a range of reward magnitudes (Figure 7E), which would result in dopamine release to fully-predicted reward. These populations therefore performed significantly worse at state-value estimations than DMS and NAc core. Taken together, this suggests that populations projecting to medial regions of the striatum (DMS and NAc core) might convey dopamine signals that are tuned to support state-value learning, whereas populations projecting to lateral regions (VLS and DLS) might be less well suited to this role.

## Discussion

Here, we defined at millisecond resolution the behavior-related activity of individual, precisely localized, dopamine neurons. In doing so, we identified considerable heterogeneity in the encoding of reward-related signals by midbrain dopamine neurons. Heterogeneity could not be well explained by anatomical subdivisions nor spatial location, whereas grouping neurons according to the striatal regions they might innervate revealed populations with divergent properties. The differential encoding of reward we observed was also evident in dopamine axons in the corresponding target regions of striatum. We show that individual dopamine neurons not only multiplex signals by encoding different combinations of egocentric and allocentric parameters, but they also exhibit different magnitudes of encoding from the rest of the population. Our TD modelling predicts that such distributional coding not only maximizes the dynamic range of dopamine signals, but also tailors them to support specialized functions of different striatal regions.

The role of dopamine neurons in predicting reward forms an important foundation for the understanding of how animals learn^39^. The dopamine signal has traditionally been considered to be uniform, broadcasting a common teaching signal across many brain circuits^3,4^. Instead, we find that such signals are far from uniform and our data suggest that different striatal regions receive specialized dopamine signals. Previous studies have identified that heterogeneity in dopamine neuron signaling can be parsed according to spatial localization in the midbrain^14,16,45^. While we cannot rule out the possibility of spatial organization, our analyses did not identify clear homogenous responses segregated by location (Figure 3); however, when we considered neurons that putatively project to the same projection targets (Figure 5), we observed more homogeneous responses. This suggests that the combination of cell body location along with molecular profiles provides a better description of cell populations than either property by itself. The anatomical location of these projection-defined groups suggests that there are “hot spots” containing neurons projecting to the same region e.g. in parts of the medial substantia nigra pars compacta (SNCM) and the VTA (Figure 5E). Indeed these “hot spots” may explain why some studies observe more uniform responses when recording sites are more localized^2,16^.

Recent work has suggested that dopamine neurons not only encode RPE, but may also encode other parameters including movement onset and kinematics^13–18,46^. In addition to neurons encoding general body movements, we identified a number of neurons that encoded licking (Figure 1D, 1E, and Figure S2). It is not clear whether these signals represent a motor or perceptual response; in principle, firing at licking could signify the initiation of a tongue movement, the sensory properties of contact with the spout, or a reward-related signal^15^. This is further complicated by the possibility that there could simultaneously be a motor response in neurons projecting to DLS but an incentive response in those projecting to the NAc core; further work will be needed to disambiguate these possibilities. Many individual dopamine neurons encoded the cue; however, in contrast to previous studies^1,47^, we did not observe a significant net response to the cue across the whole population (Figure 1C and 6A). Previous work has suggested that the cue serves an alerting role^48–53^. In support of this idea, dopamine neurons do not respond when the offset of a sound is used as a cue, they show larger responses to strong sensory stimuli, and they exhibit diminished responses to cues predicting rewards with 100% reliability^49,52,54,55^. The cue we used only had a modest volume (62 dB), which is considerably quieter than many commercial systems (which can be 75 – 86 dB). In primates, a 72 dB tone only elicited a small change in dopamine firing whereas a 90 dB tone caused a large phasic increase^55^. It is therefore possible that introducing louder cues or changing reward probabilities would unmask a larger increase in firing to the cue^15^. It has also been suggested that the cue response signals motivational salience, with higher value stimuli eliciting a larger response^51,56^. In our experiments, we used relatively mild motivation strategies^57^, and one might therefore predict that if the motivational drive of the mice were very high, there would be a larger dopamine signal to the cue^58,59^. Regardless of the explanation, one important observation from our data is that a positive dopamine response at cue presentation is not necessary for Pavlovian conditioning.

Our data suggest that dopamine neurons projecting to different regions have distinct firing patterns; we confirmed these observations by measuring distinct signals in dopamine axons in different regions of striatum. These results argue against a model^60^ where the firing of dopamine neurons is distinct from activity in dopaminergic axons, but instead support ideas that there are distinct profiles of dopamine release in different parts of striatum^5–13,30,61^. Perhaps the most striking difference between responses we observed was that the putative DMS-projecting group did not respond to predicted reward; this finding is in agreement with some studies^7,9^ but not others^5,11,30^. The fact that reward probability was deterministic in our experiments may help to reconcile these apparent discrepancies; a recent study compared dopaminergic axon terminal responses in DMS during fixed-vs variable-probability reward and observed a similar lack of response to fixed reward which was rescued as rewards became probabilistic^9^. In contrast to DMS, we observed that the VLS-projecting population responded strongly to predicted reward and that NAc core-projecting neurons responded during licking. Similar pronounced reward signals have previously been observed in dopamine neuron terminals within VLS, and delayed signals in medial regions which might be consistent with licking rather than reward^6^. Interestingly, aversive taste is reported to result in dopamine release preferentially in NAc core, suggesting a possible evaluative role for these licking-related signals^10^.

What are the implications of projection-selective-encoding? Because dopamine signals likely result in different outcomes depending on the target region (e.g. cue attraction vs movement invigoration)^38,61^, it follows that different striatal territories might receive distinct dopamine signals. Such specialized signals would permit flexibility and a wide dynamic range; for example, different regions might receive a common signal in one learning scenario for appetitive situations where approach is desired, but tailored signals in aversive scenarios where avoidance would be the appropriate behavior^6,11,30^. Our modelling suggests that responses may be tuned to different parts of the reward spectrum. For example, the positively skewed DLS- and VLS-projecting responses (Figure 7A) suggest populations of dopamine neurons which tend to underestimate reward. One might expect such patterns of dopamine release to reinforce actions which could support habit development (a role that has been previously ascribed to DLS)^62,63^. This segregation of signaling profiles could facilitate simultaneous accurate reward evaluation in medial regions and action reinforcement in lateral striatum. As such, distributional coding within discrete projection-defined populations may impart additional benefit compared with coding by a single population^9,44^. The heterogeneity we observe may also be compounded by the possibility that dopamine neuron populations projecting to different regions may co-release glutamate, GABA, or neuropeptides^29,64,65^. Furthermore, we report dopamine signals at the soma and axon, but dopamine release dynamics may be shaped locally and the striatum itself is heterogeneous with differences in dopamine transporters, cholinergic signaling, and striosome and matrix compartments across the striatum^66,67^. Further investigation is required to understand how differences in dopamine signaling interact with this additional complexity.

In conclusion, we find that even in simple learning paradigms, dopamine neurons represent multiple behavioral parameters in a heterogeneous manner. However, our data reveal an organizational logic where different striatal regions receive dopamine signals that are specialized to support different aspects of learning.

### Limitations of the study

One of the challenges of studying dopamine neuron subtypes is to fully define all existing populations. Single-cell transcriptomics has been used to identify putative dopamine neuron subsets based on expression of common sets of genes^21–26,46,68–70^ and has identified at least seven populations^26^, although it is likely that there are further subgroups^46^. In our study we attempted to identify populations based on the striatal regions they innervate. We identified combinations of marker expression and cell body location that could be used to delineate which striatal region a dopamine neuron is likely to target. While DLS- and NAc core-projecting populations exhibited “all or nothing” expression of three key markers, DMS- and VLS-projecting populations were less clear cut. This raises the possibility that there may be more than one population of dopamine neurons projecting to these regions. For example, in addition to the Sox6+ Aldh1a1-population projecting to VLS we identified, there could also be some Sox6-Aldh1a1+ dopamine neurons which are likely to target intermediate regions (i.e. between VLS and DLS) as Aldh1a1 expression decreases in ventral regions^68–70^. Similarly, some of the remaining heterogeneity within our four populations could be accounted for by the presence of additional subgroups within these populations; for example, the recently identified Anxa1-expressing subtype of dopaminergic neuron that projects toDLS^46^. We also cannot rule out the possibility that a proportion of neurons with a marker and localization profile ascribed to a striatal target region might project to another brain region^69^ (e.g. a proportion of neurons in medial SNc expressing Aldh1a1 and Sox6 could project to a region other than DMS). However, the concordance between neuronal firing and the activity of dopamine axons (recorded with photometry) in the ascribed striatal regions provides confidence that this is either rare, or these populations respond similarly during behavior.

## Star★Methods

## Resource Availability

### Lead contact

Information and requests for resources and reagents should be directed to the and will be fulfilled by the Lead Contact, Dr Paul Dodson (paul.dodson@bristol.ac.uk)

### Materials availability

This study did not generate new unique reagents.

## Experimental Model And Subject Details

### Experimental animals

All experimental procedures on animals were conducted in accordance with the Animals (Scientific Procedures) Act, 1986 (United Kingdom) and approved by the animal welfare and ethical review boards at the University of Bristol and the University of Oxford. N=47 C57Bl6/J 3 – 4 month-old male mice (Charles River Laboratories) were used for recording and tracing and N=4 DAT^IRES*cre*^ (JAX:006660) 2 – 3 month-old male mice (heterozygous for the transgene) were used for fiber photometry experiments. Mice were group housed (except when isolated to prevent fighting or for experimental needs) in open-top (Bristol) or individually-ventilated (Oxford) cages. Cages were enriched with a house, cardboard tube and wooden chew block. Mice were kept in temperature-controlled conditions (21°C) and on a 12:12-h light–dark cycle (lights OFF at 08:15, lights ON at 20:15); experimental procedures were performed during the light phase of the cycle. Standard laboratory chow (Purina, UK) and water was provided *ad libitum* (except during food or water restriction).

## Method Details

### Surgeries

Mice were anesthetized using 1 – 2% (vol/vol) isoflurane and placed in a stereotaxic frame, on a homeothermic heating mat (Harvard Apparatus) to ensure stable body temperature. Corneal dehydration was prevented using carbomer liquid gel (Viscotears, Alcon) and mice were perioperatively injected with the analgesic buprenorphine (0.03 mg/kg s.c., Vetergesic, Bayern).

For electrophysiological recordings, a custom L-shaped headpost (0.7 – 0.8 g, stainless steel or aluminum) was attached to the skull using cyanoacrylate glue^14^. The 3 mm diameter window in the headpost-base was positioned above the substantia nigra of the right hemisphere (centred at AP -3 mm and ML +1.5 mm from bregma). A craniotomy for single-unit recordings was made within the window of the headpost either on the day of headpost implantation or 1 – 7 days prior to recording. Two stainless steel screws (0.8 mm diameter; Precision Technology Supplies) were implanted in the skull, one above the frontal cortex and a reference above the cerebellum of the left hemisphere. A coiled 0.23 mm diameter stainless-steel wire (AM Systems) was implanted between the layers of cervical muscle to record EMG activity (filtered at 0.3 – 0.5 kHz). Exposed skull, screws and EMG wire were covered with dental acrylic resin (Jet Denture Repair; Lang Dental). The craniotomy was sealed with fast set removable silicone rubber (Body Double; Smooth-On).

For retrograde tracer injections, a craniotomy was performed above the target region and a calibrated glass micropipette (708707; Blaubrand IntraMark) with a tip diameter of ~25μm was lowered to the appropriate target; NAc core (AP +1.0, ML +1.0, DV -4.3), DLS (AP +1.1, ML +1.8, DV -3), DMS (AP +1.0, ML +1.2, DV -2.8), VLS (AP +1.0, ML +1.8, DV -4.2). 30 – 150 nl cholera toxin subunit b (CTB; 0.5% w/v; C9903; Sigma-Aldrich) was manually injected at a rate of ~50 nl/min and pipettes were left in place for 5 – 10 minutes after injection. 9 – 13 days after tracer injection, mice were given a lethal dose of anesthetic and transcardially perfused. In a minority of experiments (N = 8), we injected CTB into dorsomedial striatum prior to electrophysiological recording, to verify that recorded neurons projected to the putatively assigned target; in these experiments we recorded and juxtacellularly labeled two SNCM neurons, both of which were CTB positive.

### Behavioral training

Animals were head-fixed using a custom headpost holder connected to a stereotaxic frame and positioned upon a custom-made treadmill where they could run, walk, or rest at will. Mice (N=34) were trained to associate an auditory cue with the delivery of a reward in a Pavlovian conditioning paradigm using a custom Arduino-based apparatus. Trials consisted of cue presentation (1 second, 4 kHz, 62 dB) delivered by a piezo speaker (535-8253, RS components), 1 second delay, followed by reward delivery (5 μl of 10% sucrose). Inter-trial interval (ITI) durations were randomly drawn from an exponential distribution with a flat hazard function to ensure equal distribution of expectation (4 – 10 s, median 5.4). Mice were either food (to > 85% of baseline weight) or water restricted (4 hours of *ad libitum* water per day after training/recording sessions using an automated water delivery system https://doi.org/10.5287/bodleian:Vj4YaGAOY); no differences in electrophysiological responses were observed between these motivators. Animals were trained in daily sessions consisting of 100 rewards and all mice showed robust anticipatory licking to cue before recording, (licking rate > 2 standard deviation from baseline during cue; median 5 days training prior to recording, IQR 3). Licking was monitored using a piezoelectric sensor (285-784, RS components). Movement periods and licking bouts for single-unit recordings were determined for the whole recording session off-line using cervical EMG and video recordings (30 frames/s). Movement typically involved walking or running on the treadmill as well as postural adjustments. Lick-onset was defined as the first video frame with visually detectable lower jaw movement, lick-offset was defined as the first of a series of at least three subsequent video frames with no visually detectable jaw movement. Movement onset and offset were defined in the same way using body and limb movements.

### Electrophysiological recording

Extracellular single-unit recordings were made with borosilicate glass electrodes (tip diameter 1.0 – 1.5 μm, in situ resistance 10 – 25 MΩ; GC120F-10, Harvard Apparatus) filled with saline solution (0.5 M NaCl) containing Neurobiotin (1.5% w/v, Vector Laboratories). Sterile saline (0.9% w/v NaCl) was frequently applied around the craniotomy to prevent dehydration of the exposed cortex. Electrode signals were filtered at 0.3 – 5 kHz and amplified 1000 times (ELX-01MX and DPA-2FS, NPI Electronic Instruments). A Humbug (Quest Scientific) was used to eliminate mains noise at 50 Hz. All biopotentials were digitized online at 20 kHz using a Power 1401 mk3 analog-digital converter (Cambridge Electronic Design) and acquired using Spike2 software (version 7 or 10; Cambridge Electronic Design). For the recording, electrodes were lowered into the brain using a micromanipulator (IVM-1000; Scientifica). To avoid possible sampling bias, on-line criteria were applied to guide recordings of dopamine neurons (spike duration threshold-to-trough for bandpass-filtered spikes > 0.8 ms and firing rates < 20 spikes/s)^71^. Following recording, single neurons were juxtacellularly labeled with Neurobiotin^14^ to allow for their unambiguous identification and localization. At the end of the experiment, mice were given a lethal dose of pentobarbital and transcardially perfused with PBS followed by 4% w/v paraformaldehyde in 0.1 M phosphate buffer (PFA). Brains were placed in PFA overnight at 4°C and then stored in PBS containing 0.05% w/v sodium-azide.

### Immunohistochemistry

50 μm coronal sections were cut from the midbrain on a vibrating-blade microtome (VT1000S; Leica Microsystems or DTK-1000, DSK). To confirm the location and neurochemical identity of recorded and juxtacellularly-labeled neurons, sections were incubated for 4 h at room temperature in PBS with 0.3% (vol/vol) Triton X-100 (Sigma) containing Cy3-conjugated streptavidin (1:1000) (GE Healthcare). To probe expression of different molecular markers in labeled neurons, a two-step procedure was applied, sections were incubated overnight in PBS-Triton with mouse anti-Tyrosine Hydroxylase (TH, 1:1000, T2928, Sigma-Aldrich) or chicken anti-TH (1:500, ab76442, Abcam); guinea pig anti-Sox6 (1:1000, gift from M. Wegner, Friedrich-Alexander University Erlangen-Nuremberg; (Stolt et al., 2006)) or rabbit anti-Sox6 (1:500, ab30455, Abcam). Sections were washed in PBS, and then incubated in PBS-Triton for > 4 hours with AMCA-conjugated secondary antibodies (donkey anti-mouse IgG, 1:500; 715-155-150 or donkey anti-chicken IgG, 1:500, 703-155-155; Jackson ImmunoResearch) or Brilliant Violet 421-conjugated secondaries (donkey anti-chicken IgG 1:500, 703-675-155, Jackson ImmunoResearch) to visualize immunoreactivity for TH, and AlexaFluor 647- or Cy5-conjugated secondary antibody to visualize immunoreactivity for Sox6 (A647: donkey anti-guinea pig IgG, 1:500, 706-605-148, Jackson ImmunoResearch; Cy5: donkey anti-rabbit IgG, 1:500, 711-175-152, Jackson ImmunoResearch). After imaging, the second step consisted of incubating overnight in PBS-Triton with rabbit anti-Aldh1a1 (1:500, HPA002123, Sigma-Aldrich) and goat anti-calbindin (1:500, sc7691; Santa Cruz) or mouse anti-calbindin (1:500, CB300, Swant), washing in PBS and then incubating overnight at room temperature in PBS-Triton with AlexaFluor 647- or Cy5-conjugated secondary antibodies (the fluorophore used in the previous step to visualize Sox6) to visualize immunoreactivity for Aldh1a1 (AF647: donkey anti-rabbit IgG, 1:500, 711-605-152, Jackson ImmunoResearch; Cy5: donkey anti-rabbit IgG, 1:1000, 711-175-152, Jackson ImmunoResearch) and AlexaFluor 488-conjugated secondary antibodies for Calbindin (donkey anti-goat IgG, 1:500, A11055, Life Technologies; donkey anti-mouse IgG, 1:500, 715-545-150, Jackson ImmunoResearch). This way, we were able to clearly visualize immunoreactivity for Sox6 (nuclear) and Aldh1a1 (cytoplasmic) using the same fluorescence channel. Borders of VTA and SNc were delineated using Aldh1a1 and calbindin immunofluorescence^72^.

For retrograde tracing, a combinatorial approach with partial overlap was used for immunohistochemistry, so that TH and CTB immunoreactivity was tested in all samples, but different series from the same animal could be tested for three additional markers. A number of markers have been identified as being selectively expressed in populations of dopamine neurons^21–26,46,68–70^; we therefore selected the three proteins (Sox6, Aldh1a1, and calbindin) that show good population discrimination and can be reliably detected using immunohistochemistry. Sections were incubated overnight at room temperature in PBS-Triton with chicken anti-TH (1:250/500, ab76442, Abcam), mouse anti-CTB (1:500, ab35988, Abcam) or goat anti-CTB (1:5000), #703, List Biological Labs), rabbit anti-calbindin (1:1000, CB38, Swant) or goat anti-calbindin (1:500, sc7691, Santa Cruz) or mouse anti-calbindin (1:500, CB300, Swant), rabbit anti-Sox6 (1:4000, ab30455, Abcam), rabbit anti-Aldh1a1 (1:500, HPA002123, Sigma Merck). Sections were washed in PBS and then incubated for > 4 h at room temperature in PBS-Triton and secondary antibodies. To visualize immunoreactivity for TH, AMCA- or Brilliant Violet 421-conjugated secondary antibodies were used (AMCA: donkey anti-chicken IgG, 1:500, 703-155-155, Jackson ImmunoResearch; BV421: donkey anti-chicken IgG 1:500, 703-675-155, Jackson ImmunoResearch). CTB was visualized using Cy3- or AlexaFluor 488-conjugated secondaries (Cy3: donkey anti-mouse IgG, 1:500, 715-165-151, Jackson ImmunoResearch; AF488: donkey anti-goat IgG, 1:500, 705-545-147, Jackson ImmunoResearch). Aldh1a1 or Sox6 immunoreactivity was visualized using Cy5- or AlexaFluor 647-conjugated secondaries (Cy5: donkey anti-rabbit IgG, 1:1000, 711-175-152, Jackson ImmunoResearch; AF647: donkey anti-rabbit IgG, 1:500, 711-605-152, Jackson ImmunoResearch) and Calbindin with Cy3- or AlexaFluor 488-conjugated secondaries (Cy3: donkey anti-mouse IgG, 1:500, 715-165-150, Jackson ImmunoResearch; AF488: donkey anti-mouse IgG, 1:500, A-21202, Life Technologies).

### Microscopy and cell counting

Example images were acquired using a confocal laser-scanning microscope (20x objective, LSM710; Carl Zeiss, or SP8; Leica). Images for cell counting were acquired on an epifluorescence microscope (DMI6000; Leica, or AxioImage.M2; Carl Zeiss) equipped with a 20x objective. Images of the dopaminergic midbrain were acquired as a series of 21 tiles (7x,3y). Sections containing CTB positive SNc and VTA neurons with a clearly defined nucleus were counted using the ‘cell counter’ plugin on ImageJ, Fiji version 1.53q^73^ or Stereo investigator software 9.0 (MBF Bioscience). During counting, the experimenter was blind to the region targeted with CTB. To obtain percentages of midbrain dopamine neurons expressing a particular marker, counts were collapsed across sections, then divided by the number of neurons positive for both CTB and TH in each sample. Every marker-combination was counted in a minimum of three animals per striatal region. CTB injection sites in striatum were represented as honeycomb plots; a tessellated hexagonal structure was superimposed onto each image, then hexagons that were >80% by CTB immunoreactivity were coloured red at 100% opacity, opacity of hexagons that included 50 – 80% CTB immunopositivity was set at 50%. Images from each animal were superimposed and opacity was normalized.

### Fiber Photometry

DAT^IRES*cre*^ mice (JAX:006660) (N=4, 2 – 3 month-old male mice heterozygous for the transgene) were injected with AAV1-CAG-flex-GCaMP6f and AAV1-CAG-flex-tdTomato (Addgene: final titers 4.45x10^12^ and 1.475x10^12^ vg/ml respectively) into the midbrain at AP - 3.2, ML +0.5, DV -4.0 and AP -3.2, ML + 1.5, DV -4.0 relative to Bregma (~250 nl total per site). 3 weeks later, mice were implanted with a headpost (as described above), and a craniotomy was made above striatum. Mice were trained for 5 – 7 days in the Pavlovian conditioning paradigm. At the beginning of the photometry recording session, a bare fiber-terminated patch cable (200 μm diameter, 0.48 NA, Thorlabs) was lowered into the brain using a micromanipulator (IVM-1000; Scientifica: AP +1.0, ML +1.0 to +1.2 for DMS and NAc, and AP + 1.0, ML +1.8 to +2.0 for DLS and VLS; -2.3 to -2.7 from brain surface for DMS and DLS; -3.3 to -3.8 for VLS and NAc). Data are comprised of a single recording session for each striatal site in each of the four mice (i.e. one DLS, one VLS, one DMS, and one NAc core recording per mouse); only one striatal region was recorded from during each session. Sessions were conducted in a pseudorandom order (with dorsal sites recorded prior to ventral). Fiber positions were confirmed post-hoc in fixed brains by visualizing GFAP immunoreactivity (1:1000 rabbit anti GFAP; 16825-1-AP, Proteintech) surrounding the fiber track using a Brilliant Violet 421-conjugated secondary antibody (donkey anti-rabbit IgG 1:500, 711-675-152, Jackson ImmunoResearch). Photometry data were acquired at 130 Hz using a pyPhotometry^74^ board (Open Ephys). Both signals were median (5 point kernel) and low pass filtered (second order Butterworth filter with a 20Hz cut-off) and a 0.001 Hz second order high pass filter was applied to correct for photobleaching. Motion correction was performed by subtracting the best linear fit of the tdTomato signal from the GCaMP signal. Baseline was obtained by filtering the GCaMP signal with a low pass 0.001 Hz, second order Butterworth filter. The motion-corrected signal was then divided by this baseline to obtain a dF/F and each sweep normalized to 1 second before the auditory cue.

### Data analysis

Single-unit activity was isolated using template matching, principal component analysis and supervised clustering within Spike2 (Cambridge Electronic Design) and data were exported to MATLAB (Mathworks). Firing activity of labeled neurons was normalized as z-scores and used to construct peri-stimulus time histograms (PSTH; bin size 40 ms, smoothed with a 5-point Gaussian filter, half-width 70 ms) using a baseline of 1 second preceding cue onset. The first 2 principal components of the PSTHs (singular value decomposition) were used for hierarchical clustering; dendrograms were computed using an average method linkage function with Euclidean distances. To analyze which factors accounted best for changes in firing of individual midbrain dopamine neurons, a Poisson generalized linear regression model (GLM; fitglm function, MATLAB) was used to obtain a least-squares fit of the selected predictors to the recording data across the whole session. The recording session (including ITIs) was broken down into 200 ms bins of spike counts aligned to cue and reward delivery for every trial. Predictors were defined as cue, reward, licking, and movement and coded as either present or absent for every bin. Bins 0 to 400 ms from the onset of reward and cue were coded as reward and cue positive, respectively. Bins were coded as licking or movement positive if they overlapped at least 75% with licking and movement bouts, respectively. The model used a log link function and was set to predict spike counts in every bin based on the binary regressors. Deviance goodness of fit tests confirmed that firing of 78% of neurons were well fit by the GLM (P < 0.05). To determine the impact of different features, irrespective of whether they resulted in increases or decreases in firing, the absolute values of coefficients were considered; an individual cell was considered responsive to one of the four parameters if the corresponding p-value was < 0.05. Because periods of licking often occurred soon after reward delivery, we confirmed that the firing of neurons classified as ‘licking’ was time locked to lick episodes but not reward delivery (Figure S2). To analyze dopamine neuron firing properties, we used a coefficient of variability of interspike intervals (CV2) to examine firing regularity^71,75^ and robust Gaussian surprise^76,77^ to detect bursts of at least three spikes with significantly shorter ISI’s than the population of spike trains.

### Computational modelling

For each observed state, a Temporal Difference (TD) algorithm^1,78^ produced a series of value predictions (*V*_*t,i*_). Each of these Value predictions represents a single neuron (i, at time t). TD error (δ_*t,i*_) was calculated by comparing a neuron’s existing state-value prediction, with a bootstrapped (predicted) estimate of the state’s value (*V*_*t*+1,*i*_):

(1) $$\delta_{t,i}=r_{t,i}+\gamma V_{t+1,i}-V_{t,i}$$

where (*r*_*t,i*_) is the reward and *γ* the discount factor. Value predictions were updated according to the following update rule:

(2) $$\begin{array}{c} V_{t,i}\leftarrow \left(V_{t,i}+\alpha_{i}^{+}\delta_{t,i}\right)\;\;\;\;\;\;\;\;\;\;\;\;\;\;\;\;\;\text{if}\;\delta_{t,i}>0\\ V_{t,i}\leftarrow \left(V_{t,i}+\alpha_{i}^{-}\delta_{t,i}\right)\;\;\;\;\;\;\;\;\;\;\;\;\;\;\;\;\text{if}\;\delta_{t,i}<0 \end{array}$$

Where $\alpha_{i}^{+}$ and $\alpha_{i}^{-}$ are the unique positive and negative learning rates applied to each neuron (*α_i_* ~ U(0,1)). Distributional coding occurs due to each neuron converging on distinct state-value estimates – according to the balance of their positive and negative learning rates. These learning rates were randomly initialized and then fit to each neuron using a grid search. Learning rates were tailored to the projection-defined agents by minimizing the difference between δ_t,i_ at rewarded states, and a sample drawn from ‘activity distributions’ for each subpopulation. Subpopulations were approximated from neural data using a kernel density estimation.

To test these algorithms, we created an environment in which agents deterministically transition between states. At one such state, the agent receives a numerical reward randomly selected from a gaussian distribution (*r ~ N*(5,5)). Trained agents have a ‘value distribution’ associated with each state in its environment; calculated from the distribution of state-error associations across simulated neurons.

After training, agents were tested with different rewards. Agents were tested on a wider range (r ~ U (0,20)) of familiar (i.e. ~5) and larger rewards. To determine how accurate each agent was at estimating value, we calculated the mean squared error (MSE) between actual (*Y*) and predicted (*Ŷ*) value, for each cell at the rewarded state:

(3) $$MSE=\frac{1}{n}{\;}^{\sum }\;{\left(Y-\hat{Y}\right)}^{2}$$

Note that predicted value (*Ŷ*) should approximate the reward received during testing.

## Quantification And Statistical Analysis

Continuous data are presented as means with SEM, boxplots display first quartile, median and third quartile. The Shapiro-Wilk test and the Levene test were used to judge whether data sets were normally distributed with homogeneous variances (p < 0.05 to reject). For normally distributed data, a one-way ANOVA was used. If data failed normality tests, Mann-Whitney rank sum or Kruskal-Wallis one-way ANOVA on ranks with Tukey’s post-hoc method for multiple comparisons were used (MATLAB, Mathworks). Significance for statistical tests was set at p < 0.05.

## Key Resources Table

**Table:** 

REAGENT or RESOURCE	SOURCE	IDENTIFIER
Antibodies		
Mouse anti-Tyrosine Hydroxylase	Sigma-Aldrich	Cat# T2928, RRID:AB_477569
Chicken anti-Tyrosine Hydroxylase	Abcam	Cat# ab76442, RRID:AB_1524535
Guinea pig anti-Sox6	Michael Wegner; University of Erlangen-Nuremberg; Germany	Cat# Wegner_Sox6 gp, RRID:AB_2891329
Rabbit anti-Sox6	Abcam	Cat# ab30455, RRID:AB_1143033
Rabbit anti-Aldh1a1	Sigma-Aldrich	Cat# HPA002123, RRID:AB_1844722
Goat anti-calbindin D28K (C-20)	Santa Cruz Biotechnology	Cat# sc-7691, RRID:AB_634520
Mouse anti-calbindin	Swant	Cat# 300, RRID:AB_10000347
Rabbit anti GFAP	Proteintech	Cat# 16825-1-AP, AB_2109646
Bacterial and Virus Strains		
AAV1-CAG-flex-GCaMP6f	Addgene	Addgene #100835, RRID:Addgene_100835
AAV1-CAG-flex-tdTomato	Addgene	Addgene # 28306-AAV1, RRID:Addgene_28306
Experimental Models: Organisms/Strains		
Mouse: DATIREScre^(Slc6a3tm1.1(cre)B kmn)^	The Jackson Laboratory	JAX006660, RRID: IMSR_JAX:006660
Mouse: C57BL/6	Charles River	Strain code: 632
Software and Algorithms		
MATLAB 2022b	MathWorks	https://www.mathworks.com/, RRID: SCR_001622
Spike2 (version 7 and 10)	Cambridge Electronic Design	https://ced.co.uk/, RRID:SCR_000903
PyPhotometry	https://pyphotometry.readthedocs.io/en/latest/	RRID:SCR_022940
ImageJ, Fiji	http://fiji.sc/	RRID:SCR_002285
Stereo Investigator	MBF Bioscience	http://www.mbfbioscience.com/stereo-investigator, RRID:SCR_002526

## Supplementary Material

Supplemental Information

## Data Availability

All data reported in this paper will be shared by the lead contact upon request. This paper does not report original code. Any additional information required to reanalyse the data reported in this paper is available from the lead contact upon reasonable request.
