# Supplementary material for: Distributional coding of associative learning in discrete populations of midbrain dopamine neurons
Source: Cell Rep. Author manuscript; Available in PMC 2024 Jun 12. (PMC7616095; doi:10.1016/j.celrep.2024.114080)
Supplement: Supplemental Information [file EMS196652-supplement-Supplemental_Information.pdf]

SUPPLEMENTAL INFORMATION

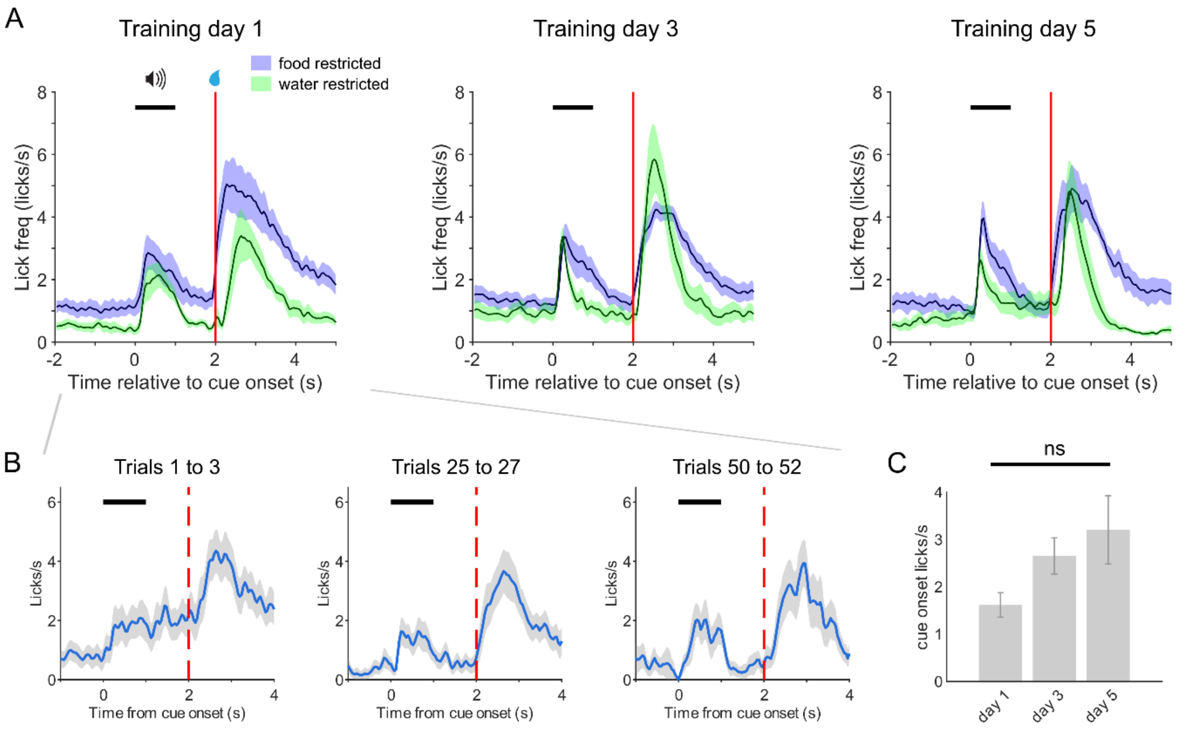

Figure S1. Mice rapidly learn to associate an auditory cue with reward delivery, Related to Figure 1. (A) Mice exhibit a Pavlovian conditioned response from the first day of training following restricted access to either food (blue) or water (green). Red line indicates reward delivery, black bar denotes the auditory cue. (B) conditioned licking rapidly evolved during the first training session on Day 1 (water and food restricted mice are pooled). (C) There was no statistical difference between lick frequency during the first half of the cue across the training days (P > 0.3 Kruskal-Wallis one way ANOVA on ranks).

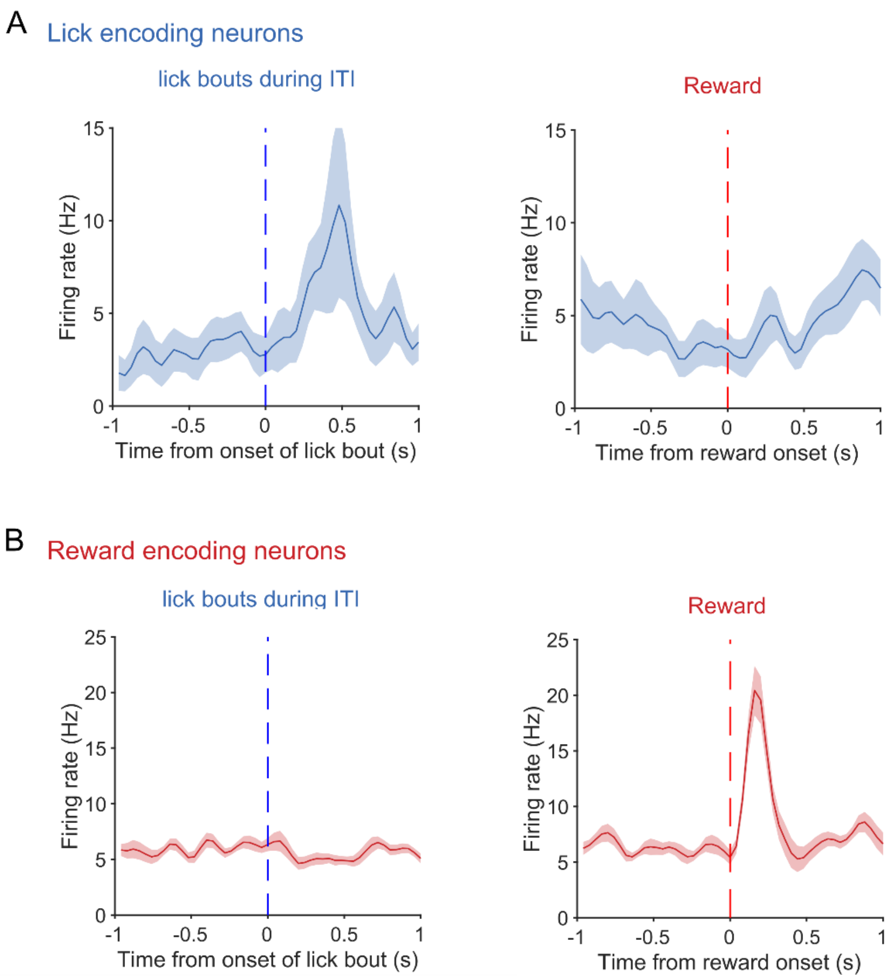

Figure S2. Increases in firing of neurons encoding licking are better matched to lick onset than reward delivery, related to Figure 1. (A) The firing rate of neurons that significantly encoded licking (but not reward) during the task also increases just after the onset of lick bouts during the inter-trial interval (blue dashed line; defined as 1.5 seconds after reward until the next cue; n = 8). (B) neurons encoding reward (but not licking; n = 12) during the task do not change their firing to licking in the ITI (left), but increase their firing as reward is delivered (red dashed line, right panel).

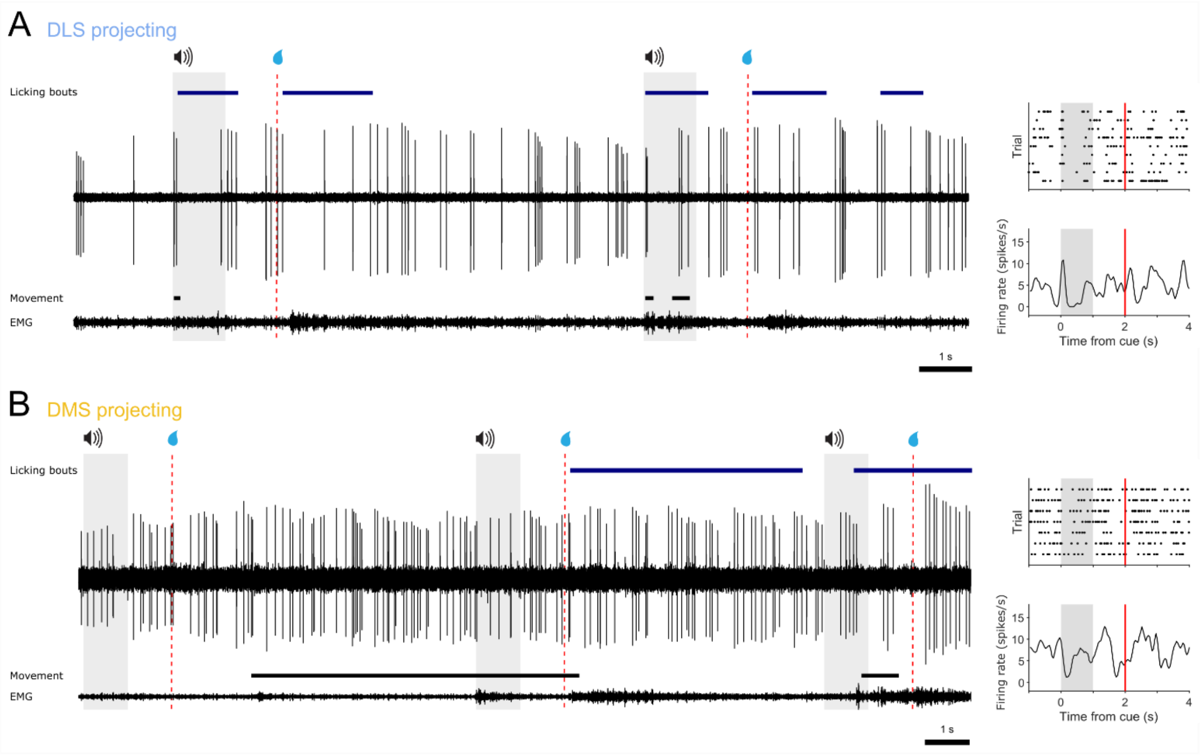

Figure S3. Firing of example dopamine neurons, related to Figure 5. Extracellular recording of action potential firing (middle), EMG recording (bottom), licking and movement bouts (blue and black bars respectively) and peri-stimulus time histogram (PSTH; right) from example DLS-projecting (A) and DMS-projecting (B) dopamine neurons during Pavlovian conditioned behavior. Grey shading indicates cue duration, red line indicates reward delivery.

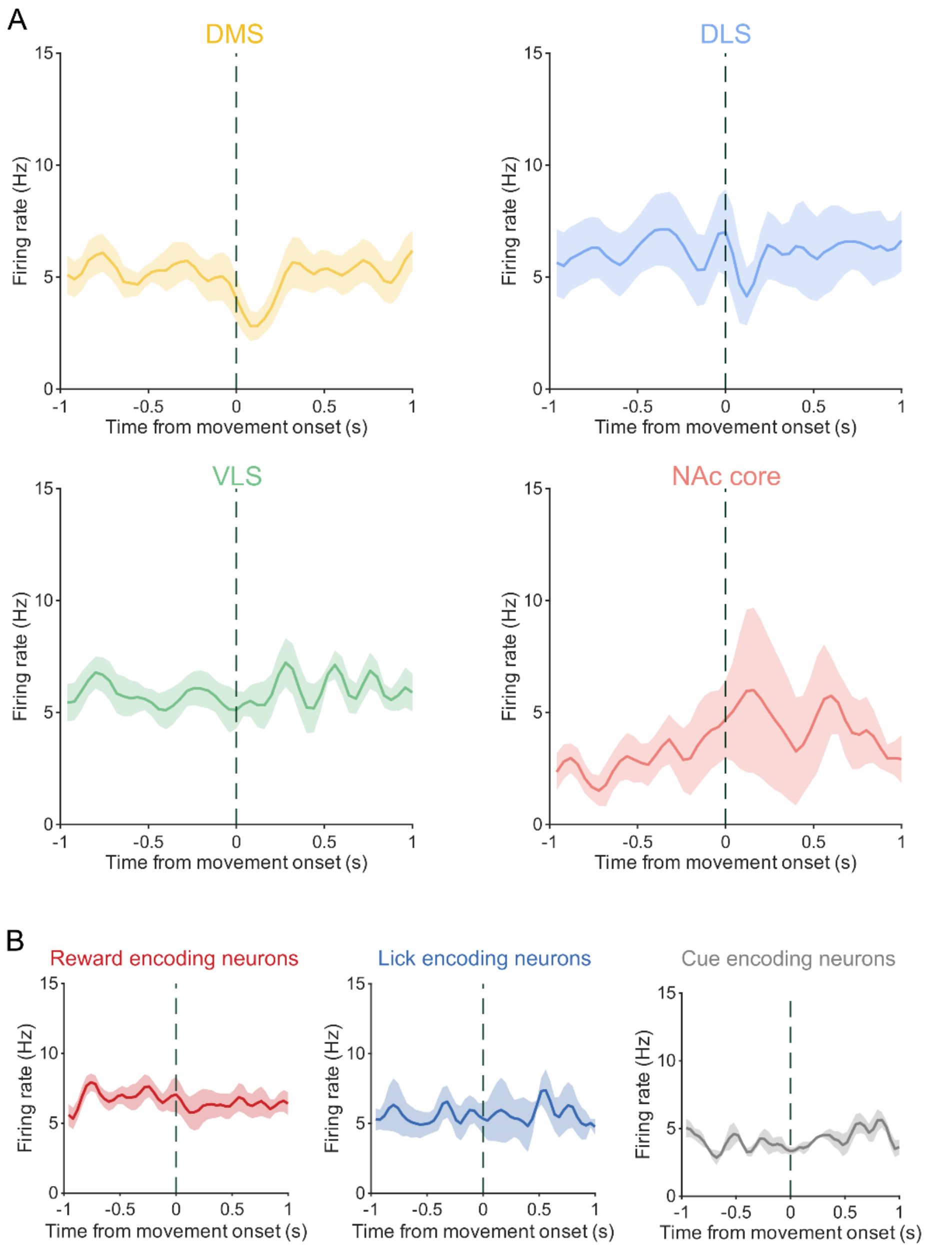

Figure S4. Firing of projection-defined populations at movement onset, related to Figure 5. (A) The firing rate of DMS and DLS-projecting populations decreases at movement onset. (B) neurons that significantly encoded reward (red; N=7), licking (blue; N=5) or cue (grey; N=5) and did not also encode movement (as identified by GLM) showed no change in firing at movement onset. Neurons in which there were no movement epochs during recording were excluded from analysis.

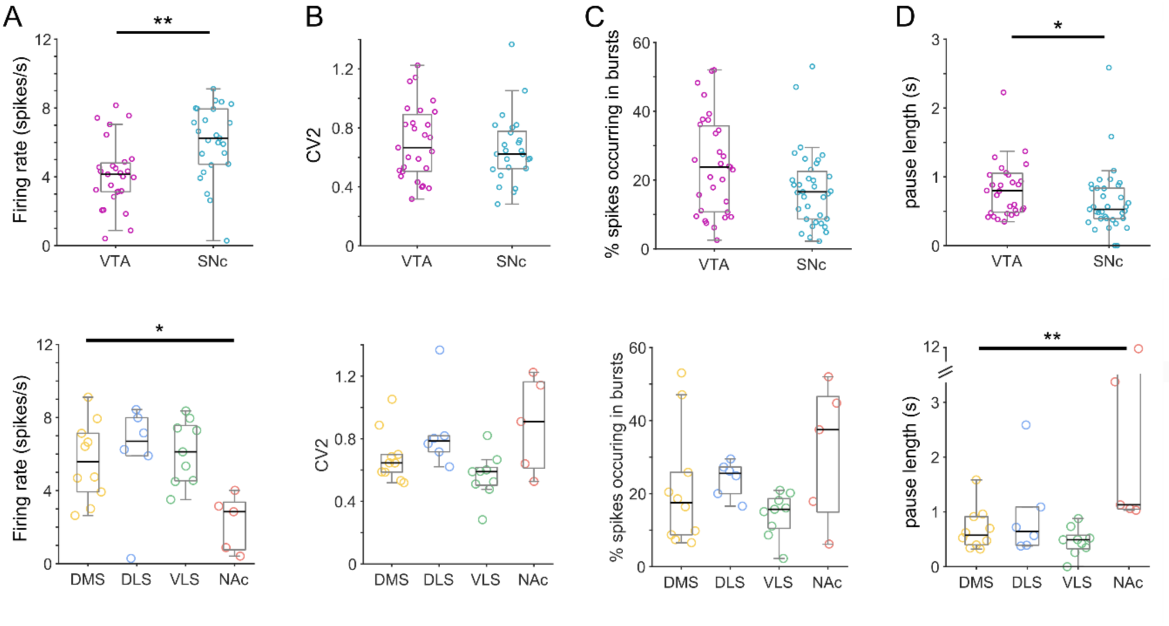

Figure S5. Distinct firing properties of dopamine neuron populations, related to Figure 5. (A) Firing rate was significantly lower in VTA neurons than SNc neurons (Mann-Whitney rank sum) and putative NAc core projecting neurons fired significantly slower than all other populations (Kruskal-Wallis one way ANOVA on ranks). There were no significant differences between neurons putatively projecting to DMS, DLS, or VLS. (B) Firing regularity (CV2) was not significantly different between VTA and SNc neurons (Mann-Whitney rank sum) nor any of the projection-defined groups (Kruskal-Wallis one way ANOVA on ranks). (C) The proportion of spikes fired as bursts were not significantly different between VTA and SNc neurons (Mann-Whitney rank sum) nor any of the projection-defined groups (Kruskal-Wallis one way ANOVA on ranks). (D) The median duration of pauses was significantly longer for VTA than SNc neurons (Mann-Whitney rank sum). Similarly, putative NAc core projecting neurons exhibited longer pauses in firing than other populations (Kruskal-Wallis one way ANOVA on ranks). * $p < 0.05$, ** $p < 0.01$.

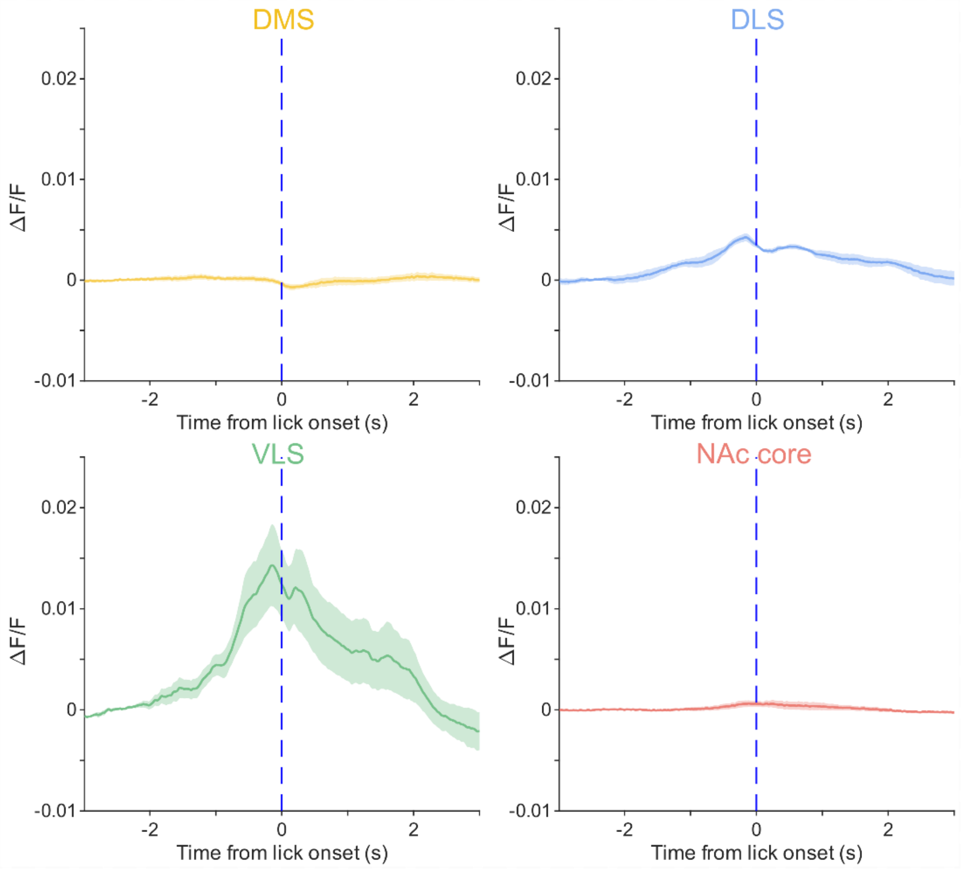

Figure S6. Lick-related signaling in different striatal regions, related to Figure 6. Mean PSTH of the change in GCaMP6f fluorescence (ΔF/F) in dopaminergic axons in DMS (yellow), DLS (blue), VLS (green), and NAc core (red) aligned to onset of all licks.

region	projection	Sox6	Aldh1a1	Calbindin	firing rate (spikes/s)	CV2	dominant feature	Cue firing (z score)	Reward firing (z score)
SNc	DLS	1	1	0	7.2	0.8	reward	2.9	11.3
SNc	DLS	1	1	0	5.9	0.8	move	1.5	1.8
SNc	DLS	1	1	0	0.3	1.4	move	0	-0.3
SNc	DLS	1	1	0	8.4	0.7	cue	0.2	0.8
SNc	DLS	1	1	0	6.3	0.8	cue	0.7	4.4
SNc	DLS	1	1	0	8.0	0.6	reward	0.2	2.3
SNCM	DMS	1	1	1	4.7	0.6	licking	0.3	1.9
SNCM	DMS	1	1	0	7.9	0.6	move	0.2	-0.6
SNCM	DMS	1	0	0	6.7	0.7	none	0.1	-0.3
SNCM	DMS	1	1	1	3.0	0.6	none	1.2	-1.5
SNCM	DMS	1	1	0	2.6	0.5	none	0.7	-1.0
SNCM	DMS	1	1	0	3.9	0.9	none	0.1	-0.3
SNCM	DMS	1	1	0	6.4	0.5	none	0.5	-0.2
SNCM	DMS	1	1	0	9.1	0.7	cue	0.5	-2.4
SNCM	DMS	0	1	0	4.7	1.1	none	0.1	-1.2
SNCM	DMS	1	0	0	7.1	0.6	none	-1.1	3.3
VTA	NAc	0	0	1	2.8	1.2	licking	-0.2	-0.3
VTA	NAc	0	0	1	3.2	0.5	none	0.6	-1.5
VTA	NAc	0	0	1	0.9	1.1	licking	-0.2	-1.0
VTA	NAc	0	0	1	4.0	0.6	licking	1.0	2.0
VTA	NAc	0	0	1	0.4	0.9	move	-0.1	1.0
PBP	VLS	1	0	0	4.5	0.5	licking	0.2	-0.8
SNc	VLS	1	0	0	8.4	0.8	reward	0.8	4.5
PBP	VLS	1	0	0	3.5	0.6	none	1.4	2.5
PBP	VLS	1	0	0	7.4	0.7	none	-0.6	-0.6
SNc	VLS	1	0	0	7.3	0.5	reward	0.2	6.8
PBP	VLS	1	0	0	4.6	0.6	reward	-0.3	6.7
SNc	VLS	1	0	0	6.1	0.6	reward	1.0	9.6
SNc	VLS	1	0	0	5.3	0.3	none	1.1	3.9
SNc	VLS	1	0	0	8.0	0.5	none	3.7	5.3
VTA	unassigned	1	1	0	7.6	1.0	none	0.8	0.0
VTA	unassigned	0	1	0	3.1	1.1	licking	0.7	3.4
VTA	unassigned	1	0	1	2.1	0.8	licking	0.5	1.2
PBP	unassigned	0	0	0	8.2	0.8	reward	0.3	7.8
VTA	unassigned	1	0	0	2.1	0.8	move	0	-0.6
SNc	unassigned	0	0	0	8.2	0.4	none	-1.4	-1.7
VTA	unassigned	NT	1	0	3.2	0.3	reward	1.6	11.1
PBP	unassigned	0	0	0	4.9	0.8	reward	2.0	5.1
VTA	unassigned	1	0	0	4.3	0.4	reward	-0.1	16.8
VTA	unassigned	1	0	1	4.0	0.9	reward	1.8	8.9
VTA	unassigned	1	1	1	5.0	0.7	reward	0.5	1.8
VTA	unassigned	0	1	0	6.4	0.8	reward	1.2	6.0
VTA	unassigned	1	1	0	1.9	0.9	none	-0.1	1.7
VTA	unassigned	1	0	0	4.3	0.5	licking	-1.1	-0.3
PBP	unassigned	0	NT	NT	4.2	0.4	none	0.6	8.1
SNc	unassigned	1	NT	NT	5.4	0.6	none	0.6	-1.6
SNc	unassigned	NT	NT	NT	6.0	0.4	reward	0.8	6.3
SNc	unassigned	0	1	0	4.3	0.4	licking	-0.3	1.5
VTA	unassigned	0	0	0	7.1	0.5	none	-0.4	8.5
VTA	unassigned	1	0	1	3.2	0.4	none	0.9	1.2
VTA	unassigned	0	0	0	4.6	0.4	none	0.5	0.5
PBP	unassigned	1	1	1	4.1	0.5	reward	5.4	19.8

Table S1. Properties of identified dopamine neurons, Related to Figure 4. Juxtacellularly labelled neurons were classified according to the location of their soma in a given midbrain region and immunoreactivity to Sox6, Aldh1a1 and/or calbindin (1 = expressed; 0 = no detectable expression; NT = not testable); this information was used where possible to assign their putative projection target. Firing rate and variability (CV2) were determined during inter-trial intervals. The dominant feature encoded was determined using a general linear model (see methods).
